# Real-world evaluation of smartphone-based artificial intelligence to screen for diabetic retinopathy in Dominica: a clinical validation study

**DOI:** 10.1136/bmjophth-2023-001491

**Published:** 2023-12-22

**Authors:** Oliver Kemp, Covadonga Bascaran, Edyta Cartwright, Lauren McQuillan, Nanda Matthew, Hazel Shillingford-Ricketts, Marcia Zondervan, Allen Foster, Matthew Burton

**Affiliations:** 1 London School of Hygiene and Tropical Medicine, London, UK; 2 University Hospitals Sussex NHS Foundation Trust, Worthing, UK; 3 Dominica China Friendship Hospital, Roseau, Dominica; 4 Moorfields Eye Hospital NHS Foundation Trust, London, UK

**Keywords:** Diagnostic tests/Investigation, Eye (Globe), Retina

## Abstract

**Objective:**

Several artificial intelligence (AI) systems for diabetic retinopathy screening have been validated but there is limited evidence on their performance in real-world settings. This study aimed to assess the performance of an AI software deployed within the diabetic retinopathy screening programme in Dominica.

**Methods and analysis:**

We conducted a prospective, cross-sectional clinical validation study. Patients with diabetes aged 18 years and above attending the diabetic retinopathy screening in primary care facilities in Dominica from 5 June to 3 July 2021 were enrolled.

Grading was done at the point of care by the field grader, followed by counselling and referral to the eye clinic. Images were then graded by an AI system. Sensitivity, specificity with 95% CIs and area under the curve (AUC) were calculated for comparing the AI to field grader as gold standard.

**Results:**

A total of 587 participants were screened. The AI had a sensitivity and specificity for detecting referable diabetic retinopathy of 77.5% and 91.5% compared with the grader, for all participants, including ungradable images. The AUC was 0.8455. Excluding 52 participants deemed ungradable by the grader, the AI had a sensitivity and specificity of 81.4% and 91.5%, with an AUC of 0.9648.

**Conclusion:**

This study provides evidence that AI has the potential to be deployed to assist a diabetic screening programme in a middle-income real-world setting and perform with reasonable accuracy compared with a specialist grader.

**WHAT IS ALREADY KNOWN ON THIS TOPIC**

- Many diabetic retinopathy (DR) algorithms have been shown to perform with high accuracy when compared with human grading, but limited evidence has been published on real-world validation of artificial intelligence (AI) for DR.

**WHAT THIS STUDY ADDS**

- The study reports on the performance of AI for DR when deployed in real-world conditions in an existing DR programme in a middle-income setting.

**HOW THIS STUDY MIGHT AFFECT RESEARCH, PRACTICE OR POLICY**

- At national level in Dominica, this study will inform policy and practice in service delivery for DR services. Globally, this study builds on the evidence in application of AI in real-world settings.

## Introduction

Diabetic retinopathy (DR) is the most common microvascular complication of diabetes mellitus. It is a major cause of vision impairment and blindness.^1^ Retinal screening and referral for treatment for those identified having DR can prevent vision loss.^2–5^ For this reason, many countries are introducing DR screening and treatment programmes.^6–8^

A recent systematic review of DR screening found that in low-income and middle-income countries (LMIC), common barriers include limited skilled human resources and lack of access to eye facilities.^9^ Use of artificial intelligence (AI) for grading of retinal images could help to reduce the time spent by ophthalmic specialists reviewing images.^10 11^ AI in DR screening can allow quick assessment of a large number of images and communication of the decision to refer, or not, to the patients at the point of care, and in the last few years these technologies have started to be validated.^12–14^ As the quality of smartphone cameras improves, there has been investment and research into their use as portable retinal cameras, offering a lower cost and transportable option in low resource and rural settings.^15^

Four recent meta-analyses reported sensitivities for AI to grade DR between 87% and 97%.^16–19^ Most studies reported AI systems which used datasets from high-quality images taken with state-of-the-art retinal cameras in eye clinic settings. Some studies, including a large-scale real-world use of AI in Thailand, have assessed community screening in field settings, reporting sensitivities between 84% and 91% for referable DR and 91% for vision threatening DR.^20–22^

The prevalence of diabetes in the adult population in Dominica is estimated to be 17.7%.^23^ Dominica has been screening for DR since 2005, but its programme coverage is limited with approximately 1500 of the estimated 7000 adults living with diabetes being screened each year. There are two employed ophthalmic technicians in the public sector in Dominica certified to grade retinal images, but their time to screen DR is limited by other clinical duties. There are two retinal cameras, one fixed (Centervue DRS) in a hospital in Roseau, the capital, and a smartphone camera (Remidio) used in a mobile clinic that visits rural districts. The ophthalmology services in Dominica are equipped to deliver treatment to patients with vision threatening DR.

AI-assisted grading in the mobile clinic could help overcome human resources constraints and increase DR screening coverage. There is an AI software application that can be used offline with the smartphone-based ‘Fundus on Phone’ retinal camera currently used in Dominica.^24^ Studies in India using this AI software and camera have reported a sensitivity of 83% to detect any DR, and a sensitivity of 93% to detect ‘referable’ DR.^25–27^

This study aimed to evaluate the diagnostic accuracy of Medios AI software for the diagnosis of referable diabetic retinopathy (RDR) using mydriatic retinal images when deployed and integrated in a real-world DR screening programme in a Caribbean population in Dominica.

## Materials and methods

### Study design

This prospective, cross-sectional clinical validation study was conducted to assess the performance of an AI software application in identifying referable DR, compared with a human grader (reference standard). The technology we tested was Medios DR AI software (NM App V.2.0, Mediostech, Singapore) hereafter referred to as ‘AI system’, incorporated into a Non-Mydriatic Fundus on Phone Camera, Model FOPNM-10, (Remidio Innovative Solutions, Bangalore, India). This AI system is Conformitè Europëenne marked and was chosen as it was compatible with the camera routinely used in the mobile programme.

The reference standard was the image grading performed in the field by the senior Dominican screener–grader, holder of a Certificate of Higher Education in DR Screening, Gloucester Retinal Education Group, University of Gloucestershire, UK (hereafter referred to as field grader).^28^ The grading by the field grader was compared with remote grading by senior graders in the English National Screening Programme, and the interobserver reliability kappa coefficient was calculated.^29^

### Participants and setting

A consecutive sample of patients with diabetes over the age of 18 years attending the mobile DR screening clinic in Dominica from 5 June to 3 July 2021 was enrolled in the study. Screening was conducted in primary care health facilities in four health districts. Informed consent was obtained from all participants. There was no change to normal practice in the screening programme clinical pathway.^30^

### Image acquisition and grading

Following the local protocol, the pupils of patients were dilated (tropicamide 0.5% and phenylephrine HCL 5%). A minimum of one image centred on the optic disc and one image centred on the macula were taken of each eye using the hand-held camera by the field grader. The field grader performed DR grading and decided to refer or not based on the grading. Patients received the usual standard of care, which includes counselling on diabetes control and referral to the eye clinic.

Although the AI system can work offline and therefore potentially provide a point of care decision, in this validation, study AI grading was deferred to the end of the study to ensure that any AI output did not influence grading and clinical decisions about referral.^27^

### Analysis

RDR was defined as moderate non-proliferative diabetic retinopathy or worse, or diabetic macular oedema, or ungradable image in either eye. Sensitivity, specificity with 95% CIs and area under the curve (AUC) were calculated for RDR comparing the AI system to field grader as gold standard. Vision-threatening diabetic retinopathy (VTDR) was defined as the presence of proliferative diabetic retinopathy and/or diabetic macular oedema in either eye. Data were collected using electronic tablets and later converted into Excel and analysed using Excel and Stata X software.

### AI and human grading

The AI system is based on convolutional neural networks and its functionality has been described in detail elsewhere.^27^ The AI provides a binary output of ‘signs of DR detected’ or ‘signs of DR not detected’ with a threshold of ‘moderate non-proliferate DR’ and above, according to the International Classification of Diabetic Retinopathy (ICDR).^31^

The field grader has been trained on, and uses, the English Grading System for DR.^6^ This system does not correspond directly with the ICDR. The lower grade of DR, referred to as R1 in the English system is equivalent to both ‘mild and moderate non-proliferative DR’ in the ICDR. To allow comparability in the study, we asked the field grader to record retinal DR features in all mild and moderate cases and subsequently classified images accordingly.

### Ungradable images

We defined ungradable images as those reported as such by the field grader. The AI system does not report an ungradable category, rather it performs a quality assessment for each image and notifies the user if the image is low quality and prompts a recapture of the image.^27^ This gives the technician the chance to retake the image until the AI quality threshold is achieved. This functionality was not used in the study, as we did not use the AI in the field to avoid introducing bias with the field grader. As the AI system actually produces a grade output for every image, regardless of the quality, we obtained AI grades for all images in this study, but in the analysis excluded AI reports for patients which the field grader reported as both eyes being ungradable.

### Sample size

Based on previous validation studies, we assumed that the AI system would have an estimated sensitivity of 93% and a specificity of 89% for detecting moderate non-proliferative DR or worse, the threshold used in our definition of referable DR.^25–27^ We also estimated that 3 in every 10 patients screened in the programme require referral to the diabetic eye clinic based on previous Dominica data; this is consistent with the expected prevalence of DR in people with diabetes.^32^ Our sample calculations, with a margin of error of 5%, gave for sensitivity sN=333 and for specificity spN=461. We took the largest estimate and added 46 participants to account for an estimated 10% ungradable cases leading to a total minimum sample of n=507.^33^

## Results

Our study included 587 participants, with a mean age of 64 years (range 26–94); 426 (72.6%) were women (table 1). The predominant ethnicity was black Caribbean (570, 97.1%). A total of 2327 images were obtained from these 587 participants. The field grader classified 72 participants in the study as having ungradable images in at least one eye (72/587, 12.2%), of which 52 had ungradable images in both eyes (52/587, 8.8%). The interobserver agreement between the field and remote image graders for detecting any DR was K=0.69 (good agreement 0.61–0.80).

**Table 1:** Participant characteristics (n=587)

Age (years)	Mean (SD)	64 (12.3)
	Range	26–94
Gender	Women	426 (72.6%)
	Men	161 (27.4%)
Ethnicity	Black Caribbean	570 (97.1%)
	Carib	17 (2.9%)
Years lived with diabetes*	Mean (SD)	12 (8.8)
	Range	0–49
Methods of diabetes control	Diet and exercise only	5 (0.9%)
	Tablet medication	517 (88.1%)
	Insulin	100 (17.0%)
	Insulin and tablet	54 (9.2%)
Type of diabetes	Type 1	6 (1.0%)
	Type 2	581 (99.0%)
Field grader DR grading	RDR	267 (45.4%)
	VTDR	111 (18.9%)
	One eye ungradable	20 (3.4%)
	Both eyes ungradable	52 (8.8%)

*n=549, some missing data for years lived with diabetes.

DR, diabetic retinopathy; RDR, referable diabetic retinopathy; VTDR, vision-threatening diabetic retinopathy.

The prevalence of RDR (moderate non-proliferative diabetic retinopathy or worse or diabetic macular oedema), including all participants (n=587), was 45.4% (95% CI, 41.5% to 49.5%) by the field grader and 39.8% (95% CI, 35.9% to 43.8%) by the AI system. The prevalence of RDR in the sample, excluding the ungradable participants (n=535), was 40.1% (95% CI, 36.0% to 44.3%) by the field grader and 37.7%% (95% CI, 33.6% to 41.9%) by the AI system.

For all participants, including ungradable images, the AI system had a sensitivity of 77.5% and specificity of 91.5% compared with the field grader for detecting RDR. The AUC was 0.84 (table 2).

**Table 2:** Grading comparison between AI system and field grader, including ungradable participants

Field grader								
AI system			Not referable		Referable		Total	
	Not referable		293		60		353	
	Referable		27		207		234	
	Total		320		267		587	
	Sensitivity (95% CI)	Specificity (95% CI)		PPV (95% CI)		NPV (95% CI)		AUC
Referable or not	77.5% (72.0% to 82.3%)	91.5% (87.9% to 94.3%)		88.4% (84.1% to 91.7%)		83.0% (82.0% to 87.9%)		0.84

AI, artificial intelligence; AUC, area under the curve; NPV, Negative Predictive Value; PPV, Positive Predictive Value.

Excluding the 52 participants deemed ungradable by the field grader resulted in the AI system having a sensitivity of 81.4% and a specificity of 91.5%, with an AUC of 0.96, for detecting RDR (table 3).

**Table 3:** Grading comparison between AI system and field grader, excluding ungradable participants (n=52)

Field grader								
AI system			Not referable		Referable		Total	
	Not referable		293		40		333	
	Referable		27		175		202	
	Total		320		315		535	
	Sensitivity (95% CI)	Specificity (95% CI)		PPV (95% CI)		NPV (95% CI)		AUC
Referable or not	80.4% (75.5% to 86.3%)	91.5% (87.9% to 94.3%)		86.6% (81.7% to 90.3%)		87.9% (84.6% to 90.6%)		0.96

AI, artificial intelligence; AUC, area under the curve; NPV, Negative Predictive Value; PPV, Positive Predictive Value.

The analysis comparing the remote graders with the AI, excluding 65 participants deemed ungradable by the remote graders resulted in a sensitivity, specificity of 83.7% and 83.7% and AUC of 0.86 (table 4).

**Table 4:** Grading comparison between AI system and remote grader, excluding ungradable participants (n=64)

Remote grader								
AI system			Not referable		Referable		Total	
	Not referable		324		22		346	
	Referable		63		113		176	
	Total		387		135		522	
	Sensitivity (95% CI)	Specificity (95% CI)		PPV (95% CI)		NPV (95% CI)		AUC
Referable or not	83.7% (75.6% to 90.4%)	83.7% (75.6% to 90.4%)		93.6% (88% to 98%)		64.2% (54.5% to 73.5%)		0.86

AI, artificial intelligence; AUC, area under the curve; NPV, Negative Predictive Value; PPV, Positive Predictive Value.

The prevalence of VTDR, (proliferative diabetic retinopathy and/or diabetic macular oedema) by the field grader in the entire sample was 18.9% (95% CI 15.7% to 22.1%) and excluding ungradable participants (n=52) it was 20.7% (95% CI 17.3% to 24.2%). In the sample excluding ungradable participants, the AI system had a sensitivity of 89.2% (95% CI 82.8% to 95.2%) for detecting the presence of VTDR (which it classified as ‘signs of DR detected’). The specificity of detecting VTDR could not be calculated as the AI system only gives a binary output for DR. There were 12 participants identified as having VTDR by the field grader, but not identified by the AI system. None of the 12 had proliferative diabetic retinopathy, all were graded as having diabetic maculopathy by the field grader. On further scrutiny of these 12 images, 7 had other macular pathology, which resulted in the field grader referring. If these were excluded from the analysis, the sensitivity of the AI increases to 95.2% (95% CI, 90.7% to 99.3%).

## Discussion

A good screening test for diabetic retinopathy should ideally have a sensitivity higher than 80% and a specificity higher than 95%.^6 34^ Our study demonstrated a sensitivity and specificity for the AI system of 77.5% and 91.5% when ungradable participants were included, and 80.4% and 91.5% when participants deemed ungradable by the field grader were excluded.

The analysis excluding ungradable participants probably gives the more reliable indication of the actual performance of the AI algorithm compared with the field grader. The AI system when used in the field prompts for a repeat image if the quality is low. To avoid bias, we could not use this feature during the study and therefore we run the AI in all images irrespective of quality.

At programme level however, it is important to consider all ungradable images as by definition those patients will need to be examined by an ophthalmologist and may have corneal pathology or cataract which results in poor retinal images.

The prevalence of DR (moderate non-proliferative diabetic retinopathy or worse or diabetic macular oedema) among our study participants was 40.1% (field grader) and 37.7% (AI system). This is similar to the estimated prevalence of DR for North America and the Caribbean region of 38.1%.^32^ The regional estimates indicate 7.8% of people with diabetes have VTDR and are therefore at risk of vision loss if not treated. In our study participants, the prevalence of VTDR was 20.7%, significantly higher than the current regional estimates. The mean years living with diabetes in the study sample is quite high (12 years) and this may differ from the population-based studies included in regional estimates. Another explanation is that the higher prevalence found may indicate late diagnosis or poor diabetes control. Also, the prevalence of obesity and hypertension in Dominica is high, possibly compounding the higher progression to VTDR of our study population.^23^

This study was conducted in a real-world outreach mobile programme. The sensitivity values are below those previously reported in the literature for Medios AI (93%–100%).^25–27^ A recent review of AI software used for DR screening found sensitivities ranging from 86% to 100% for detecting ‘referable DR’, with most of these using the same definition for referable DR as our study.^10^ It is important to point out that, although the study was not powered to detect VTDR, there were 12 cases where the grader classified patients as VTDR, due to suspected maculopathy, that were not identified by the AI system, giving a sensitivity for VTDR of 89%. This reflects the fact that field graders in real-world programmes make decisions on referral of other pathology that they find while screening. In this case, seven participants had non-DR macular signs that prompted referral which the AI is not trained to pick up. An adequately powered large scale field validation of AI in Thailand achieved a sensitivity for identifying VTDR of 91.4% and reported that most of the discrepancies were related to the grading of diabetic maculopathy.^22^ When we remove the seven referrals with non-DR macular changes from the analysis, the sensitivity of the AI for VTDR increases to 95.2%.

The balance of sensitivity and specificity is very relevant at programme level. A low specificity would imply too many patients being unnecessarily referred to the eye clinic, overloading the services. The specificity of the AI system in our study was quite high, which suggests the appropriateness of the referrals made. The programme guidelines in Dominica have a low threshold for referral, with mild forms of DR being referred to the eye clinic. This is because there is no robust system for annual recall of diabetic patients for an eye examination. Referring less severe cases of DR gives an opportunity for patient education about diabetes and hypertension control and ensures the patients are registered a in the eye clinic which facilitates regular review. The threshold for referral varies from country to country and is determined by local guidelines for DR management.^35–38^ With the current programme referral thresholds, the AI system resulted in a postive predictive value (PPV) of 88.4% and 85.4% (including and excluding ungradable images in the analysis).

Our study had a women-to-men ratio of 3.5:1. Although it is reported that women are more likely to have diabetes than men in Caribbean populations, the WHO STEPwise approach to surveillance survey (STEPS) data for Dominica in 2008 showed a higher prevalence of diabetes in men.^23 39^ It is plausible that this has changed in the last decade in Dominica. An alternative explanation is that women may be accessing diabetes services more than men and are therefore overrepresented in the DR screening programme. If this is the case, it will be important to explore the reasons for the lower uptake of screening by men and implement strategies to improve it.

This study reports the performance of an AI system fully integrated in a functioning DR screening programme in an LMIC. It provides evidence that an AI system with off-line capabilities has the potential to be deployed in a mobile community DR screening programme and perform with reasonable accuracy compared with a trained specialist grader. In order to leverage the contribution of AI technology to improve DR screening coverage and address the specialised human resource constraints, it is recommended as a next step to research the performance of the smartphone camera and AI system in the hands of trained community nurses.

## Data Availability

No data are available.
